# Acupuncture and its effect on cytokine and chemokine profiles in seasonal allergic rhinitis: a preliminary three-armed, randomized, controlled trial

**DOI:** 10.1007/s00405-022-07335-5

**Published:** 2022-03-17

**Authors:** Donata Gellrich, Florian Pfab, Miriam Ortiz, Sylvia Binting, Benno Brinkhaus, Moritz Gröger

**Affiliations:** 1 Department of Otorhinolaryngology, Head and Neck Surgery, University Hospital, LMU Munich, Munich, Germany; 2 Department of Dermatology and Allergology, Technische Universität München, Munich, Germany; 3 Medical Center Residenz, Residenzstraße 9, Munich, Germany; 4 Charité – Universitätsmedizin Berlin, corporate member of Freie Universitält, Berlin, Humboldt-Universität zu Berlin, and Berlin Institute of Health, Institute of Social Medicine, Epidemiology, and Health Economics, Campus Charité Mitte (CCM), Berlin, Germany

**Keywords:** Acupuncture, Chemokines, Cytokines, Nasal secretion, Seasonal allergic rhinitis

## Abstract

**Purpose:**

Numerous studies have demonstrated effectiveness for acupuncture in the treatment of seasonal allergic rhinitis (SAR). However, the underlying mechanism remains still unclear.

**Methods:**

29 SAR patients were recruited from a large randomized, controlled trial investigating the efficacy of acupuncture in SAR. 16 patients were treated by acupuncture plus rescue medication (RM, cetirizine), 6 patients received sham acupuncture plus RM and 8 patients RM alone over 8 weeks. Patients were blinded to the allocation to real or sham acupuncture. At baseline and different time-points during intervention, plasma and nasal concentration of mediators of various biological functions were determined in addition to validated disease-specific questionnaires.

**Results:**

The concentration of biomarkers related to the Th1-, Th2-, and Treg-cluster was not changed in patients who received acupuncture, in neither plasma nor nasal fluid. However, with respect to eotaxin and some unspecific pro-inflammatory cytokines (IL-1b, IL-8, IP-10, MIP-1b, MCP-1), acupuncture led to a, partially significantly, lower nasal concentration than sham acupuncture or RM. Furthermore, the nasal symptom score was significantly reduced in patients only after real acupuncture.

**Conclusion:**

In SAR, acupuncture reduces the intranasal unspecific inflammation, but does not seem to act immunologically on the Th1–Th2-imbalance.

**Supplementary Information:**

The online version contains supplementary material available at 10.1007/s00405-022-07335-5.

## Introduction

Acupuncture, as a complementary and integrative medicine therapy, aims to target certain specific acupoints to improve the treatment of various diseases [1]. As acupuncture is a rather safe therapy, many patients try to alleviate various symptoms through acupuncture. Among patients with allergic rhinitis (AR), the estimated lifetime prevalence of acupuncture use even ranges at about 19% [2].

Numerous reviews and meta-analysis have demonstrated effectiveness for acupuncture in the treatment of both seasonal and perennial AR [3–5]. Due to its lasting and stable efficacy in alleviating AR symptoms, acupuncture is widely used with this indication not only in China, but all across the world [6]. As a consequence, acupuncture is even included as a therapy option in the United States’ current clinical guideline for ENT diseases [7].

Despite a variety of high-quality trials confirming efficacy of acupuncture in AR [8–12], acupuncture is often criticised as “unproven” [13], as strong evidence of the underlying mechanism is still missing. Most studies focus on semi-objective variables as rescue medication use or symptom scores or disease-related quality of life. Objective parameters such as changes in cytokine levels are mainly investigated in animal studies [14–17]. In the literature, only very few trials can be found analysing the molecular basis of acupuncture in AR in humans. One study on differential gene expression in the peripheral blood of patients with AR before and after acupuncture treatment suggest that the balance between T-helper 1 (Th1) and T-helper 2 (Th2) cell-derived pro-inflammatory versus anti-inflammatory cytokines might be improved by acupuncture treatment [18]. However, no control group was included in this study. In another trial, some interleukin (IL) titers were determined demonstrating a tendency of an increasing IL-10 value in the acupuncture group vs. an antihistamine treated group [19]. Although this result indicates the probability of an immunomodulatory effect [19], the evidence is limited due to a missing sham-acupunctured control group. To our knowledge, there is only one randomized, sham-controlled trial of acupuncture for persistent allergic rhinitis in adults investigating a possible modulation of mucosal immune responses [20]: after acupuncture, allergen specific IgE for house dust mite and the pro-inflammatory substance P were down-regulated, whereas all other measured mediators remained stable in saliva and plasma. Nasal secretion, which could reflect changes in mucosal immune response better than saliva, was not analysed.

As acupuncture—despite its positive clinical evidence—will only be fully accepted as treatment of allergic diseases, if a specific mechanism is proved [21], we initiated the present sub-study of a large three-armed randomised controlled trial of *acu*puncture in *s*easonal *a*llergic *r*hinitis (ACUSAR) which had shown a positive outcome of acupuncture compared to sham acupuncture and to rescue medication alone [9, 22]. The aim of the sub-study was to investigate the immediate and prolonged immunomodulatory effects of acupuncture in seasonal AR by determining various mediators in nasal fluid and plasma.

## Patients and methods

The underlying data of the present study originate from the ACUSAR trial, a three-armed, randomised, controlled multicenter study on the efficacy of acupuncture in seasonal allergic rhinitis (SAR) with regard to rhinitis-related quality of life and rescue medication score. Further details of the study protocol and the results have been published previously [9, 22]. In a sub-study, which was performed at the study center at the Technical University Munich, blood and nasal discharge was taken to investigate the effect of the interventions on various cytokine and chemokine profiles.

The ACUSAR trial as well as the present sub-study were conducted in accordance with the Declaration of Helsinki Good Clinical Practice guidelines. Written informed consent was provided by all study participants. The study protocol of the ACUSAR trial and of the present sub-study was authorised by the ethics review committee of the Technical University Munich.

### Study population

The inclusion criteria were patients aged 16–45 years, suffering from moderate to severe SAR (according to ARIA criteria) lasting for at least 2 years. Sensitization towards birch or grass had been proven; in cases of doubt with regard to the clinical relevance of sensitization, nasal allergen challenges have been performed. The exclusion criteria were perennial AR, allergic asthma, a history of anaphylactic reactions, moderate to severe atopic dermatitis, autoimmune disorders, severe chronic inflammatory diseases, hypersensitivity to cetirizine or related drugs, specific immunotherapy during the last 3 years or planned in the next 2 years, pregnancy or breastfeeding, previous acupuncture treatment for SAR, and any further use of complementary and alternative medicine.

### Randomisation and interventions

In the beginning of the pollen season and after the appearance of first SAR symptoms, the patients started to participate in the study. For randomisation, a 2:1:1 allocation ratio was used and carried out through a centralised telephone randomisation procedure. The patients receiving acupuncture were blinded to the treatment allocation (acupuncture or sham acupuncture) for the entire study.

The patients of the acupuncture group received in sum 12 sessions of semi-standardized acupuncture, 2 sessions per week during the first 4 weeks and one session weekly during the further 4 weeks of study. The patients were treated by trained physicians with additional extensive acupuncture training at four obligatory basic Chinese medicine acupuncture points (LI4, LI11, LI20 bilaterally and *Yintang*), at least three of eight facultative basic points (*Bitong*, GB20, LR3, LU7, ST36, SP6, TE17 or BL13) and at least three additional points. The patients of the sham acupuncture group were treated according to the same time protocol, however, they received superficial needling without provoking the Deqi-sensation or at pre-defined non-acupuncture points without any link to the disease.

The rescue medication (RM) group did not receive any acupuncture. All patients, regardless of their allocation, were allowed to take a second-generation oral antihistamine (cetirizine) up to twice daily. Further details are described in the study protocol previously [22].

### Data collection

Nasal fluid was collected at baseline (before the first acupuncture session), directly after the first acupuncture session, at week 4 (day 28) and week 8 (day 56). Blood samples were taken at baseline, on day 28 and on day 56.

For collecting of nasal secretion, the cotton wool method as described by Rasp et al. [23] was carried out with minor modifications according to Kramer et al. [24]. Nasal fluid was gained by introducing small cone-shaped cotton wool pieces (absorbent cotton, Hartmann, Heidenheim/Brenz, Germany) with a length of about 30 mm and a diameter of about 4–6 mm into the middle meatus of the nose. After 20 min, the cotton wool pieces were taken out and subsequently centrifuged (+ 4 °C, 3000* g*) on a sieve for 10 min. The hereby gained nasal secretions were frozen at − 18 °C.

Furthermore, within the scope of the ACUSAR study, patients reported on changes in their symptoms and medication need which was assessed in a standardized manner using the Rhinitis Quality of Life Questionnaire (RQLQ) and the Rescue Medication Score (RMS).

### Biochemical and immunological methods

After dilution to 1:5, nasal fluids were analysed for the concentration of various, below-mentioned cytokines using a human cytokine 27-plex panel according to manufacturer`s instructions (Bio-Plex Pro Human Cytokine Standard 27-Plex, Group I, Bio-Rad Laboratories, Hercules, California, USA). This assay uses fluorescently labelled polystyrene beads which are conjugated to capture antibodies directed to the cytokines. After washing, the fluorescently addressed detection antibody forms an immunoassay with the cytokine. For analysis, the fluorochromes are excited by two lasers: one for classifying each bead, the other for quantifying the amount of bound substrate [25]. The same procedure was performed with plasma, however, after dilution to 1:4. The detection threshold was 0.5 pg/ml. The determined cytokines were eotaxin as eosinophilic marker, IL-4, IL-5 and IL-13 as part of the Th2-cluster, IL2, IL-12 and IFN-gamma as markers of the Th1-cluster, IL-10 as representative of the Treg-Cluster, and unspecific inflammatory markers such as IL-1β, IL-2, IL-6, IL-7, IL-8, IL-17, TNF-alpha, MCP-1, MIP-1β and IP-10.

### Statistical analysis

Statistical analysis was performed with SigmaStat (Jandel Corp., San Rafael, CA, USA). For descriptive statistics, we used median values with range due to the small sample sizes.

Almost all data failed normality testing (Shapiro–Wilk); therefore, non-parametric tests have been used: The Mann–Whitney rank sum test was carried out to compare concentrations between different cohorts at the same time point (inter-group comparison). The Friedman analysis was used to compare the concentration at different time-points among one study group (intra-group comparison). A *p*-value ≤ 0.05 was judged significant.

## Results

### Characterization of the study population

29 patients meeting the above-mentioned inclusion criteria were included in this sub-study, which was performed at the Technical University Munich in cooperation with the medical center of the University Charité Universitätsmedizin Berlin. According to allocation ratio 2:1:1 of the ACUSAR trial, 15 patients received real acupuncture plus RM, 6 patients sham acupuncture plus RM and further 8 patients RM alone. The seventh patient receiving sham acupuncture plus RM within the ACUSAR study refused the participation in the sub-study.

Demographic and clinical data of the three study cohorts are given in detail in Table 1, separated according to the treatment arm of acupuncture plus RM, sham acupuncture plus RM and RM alone. Concerning most parameters, the three groups were well comparable.

**Table 1:** Demographic and clinical characteristics of all three treatment groups of the ACUSAR sub-study

Characteristics	Acupuncture (*n* = 15)	Sham-acupuncture (*n* = 6)	Rescue medication (*n* = 8)
Gender			
Male	8 (53%)	2 (33%)	4 (50%)
Female	7 (47%)	4 (67%)	4 (50%)
Age (yrs)	38 (27–45)	31 (18–40)	32 (23–38)
Duration of SAR [years]	20 (8–41)	22 (7–41)	24 (18–36)
SAR symptom score			
In the preceding year	60 (40–75)	68 (48–77)	60 (55–70)
In the preceding week	60 (20–97)	56 (2–83)	53 (14–86)
Oral allergy syndrome	7 (47%)	2 (33%)	4 (50%)
Asthmatic complaints	0 (0%)	0 (0%)	0 (0%)
Mild atopic dermatitis	0 (0%)	0 (0%)	1 (13%)

Age, duration of SAR and SAR symptom score are given as median and range. All other values are number of patients total and percent of each evaluated subgroup

### Concentration of biomarkers

In plasma as well as in nasal secretion, the concentration of many determined biomarkers was below the cut-off value (IL-2, IL-4, IL-5, IL-10, IL-12 and IL-17). For IL-13, almost all measured concentrations were below the cut-off value. Therefore, no meaningful conclusion could be drawn with regard to a potential effect on these markers by acupuncture.

Concerning all other measured cytokines, we found levels above the cuff-off-level, at least in nasal secretion. In general, the concentration of most cytokines was significantly higher locally in nasal fluid than measured systemically in the plasma (concentration in plasma vs. in nasal secretion: median p value 0.002, range from < 0.001 to 0.310).

### Concentration of biomarkers in plasma

Regardless of the concerned mediator and its baseline level in plasma, we did not register any statistically significant changes in plasma concentration within one treatment arm at different time points during intervention. Data are given in the Supplement (Suppl. Table 1).

Due to the large individual variation range of cytokine concentrations, the median change from baseline concentration was used for inter-group comparison: However, no statistically significant difference could be observed between the three treatment groups, which is displayed in the Supplement (Suppl. Table 2).

Thus, no intervention significantly affected the plasma level of any of the analysed biomarkers, regardless of its biological function.

### Concentrations of biomarkers in nasal secretion

Similarly, as in plasma, no clear trend could be observed locally in nasal concentrations of biomarkers related to the Th1-, Th2-, and Treg-cluster. For some cytokines belonging to these clusters, nasal concentrations were below the cut-off (IL-4, IL-5, IL-12, IL-10). In those cytokines of which the level was above the cut-off (e.g. IL-13 as Th2-marker or IFN-gamma as Th1-marker), no relevant consistent change could be found while intervention in any treatment arm.

In the group of unspecific pro-inflammatory mediators, IL-2 and IL-17 remained below the cut-off, whereas TNF-alpha and IL-6 had values above the cut-off, but did not show any uniform trend of change in concentration throughout intervention.

**Fig. 1:**
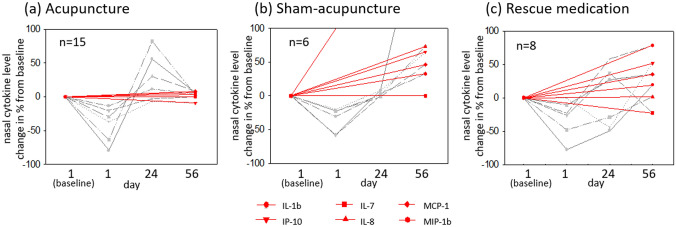
Percentage change from baseline nasal concentration of various pro-inflammatory cytokines throughout intervention consisting of **a** acupuncture, **b** sham acupuncture and **c** rescue medication. The red graphs show the change of nasal mediator level from baseline to the end of intervention. Patients treated by sham acupuncture (**b**) or rescue medication (**c**) experienced an increase in nasal concentration of most pro-inflammatory mediators. Patients treated by real acupuncture (**a**), however, had a rather equal nasal concentration of most inflammatory cytokines at the end of treatment. The grey graphs show the course of each individual analysed cytokine, which appears rather uniform in patients undergoing real acupuncture—with an initial decrease after the first session of acupuncture, followed by an increase and finally a reduction to near baseline

However, there was a similar trend with respect to some other biomarkers of unspecific inflammation, namely IL-1β, IL-8, IP-10 and MCP-1, and less pronounced MIP-1β and IL-7. As displayed by the red graphs in Fig. 1, the nasal concentration of most analysed pro-inflammatory mediators increased from baseline to the end of intervention in patients treated by sham acupuncture and rescue medication alone. In patients undergoing real acupuncture, however, the final nasal level of inflammatory cytokines was relatively similar to baseline values.

Furthermore, as shown by the grey graphs in Fig. 1, the course of nasal concentrations of the individual mediators resemble each other, particularly in the acupuncture group: directly after the first session of treatment, all patients had a lower nasal cytokine level than at baseline, especially patients treated with real acupuncture. This initial decrease was less frequently observed in patients receiving sham acupuncture or RM alone. Four weeks later, under ongoing therapy, the concentration of unspecific inflammatory markers increased in almost all groups, however, at discrepant amounts. At the latest time point of measurement, after 8 weeks of intervention, the nasal level of pro-inflammatory cytokines was the highest among patients with sham acupuncture, except for MIP-1β.

**Fig. 2:**
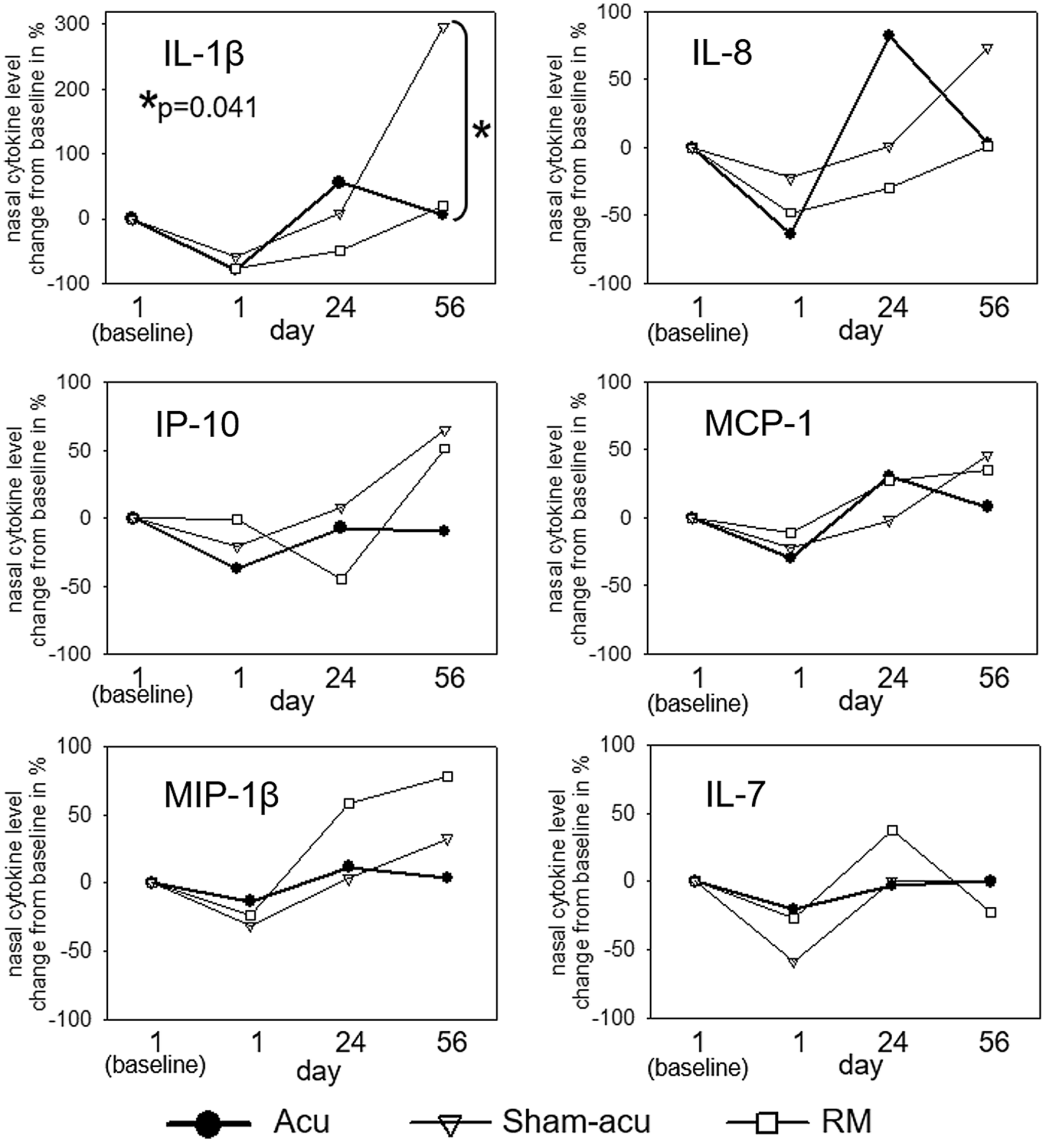
Percentage change to baseline nasal concentration of IL-1β, IL-8, IP-10, MCP-1, MIP-1β and IL-7 throughout intervention (acupuncture: black dots, sham acupuncture (white triangles) and rescue medication (white squares)

Figure 2 displays the effect of the three treatments (acupuncture, sham acupuncture and rescue medication alone) on the nasal concentration of each affected pro-inflammatory mediator.

With respect to IL-1β, IL-8, IP-10, MCP-1 and MIP-1β, the final nasal concentration was lower after acupuncture compared to sham acupuncture, even reaching statistical significance concerning IL-1β. RM led to lower pro-inflammatory cytokine levels than sham acupuncture, with the exception of MIP-1β.

**Fig. 3:**
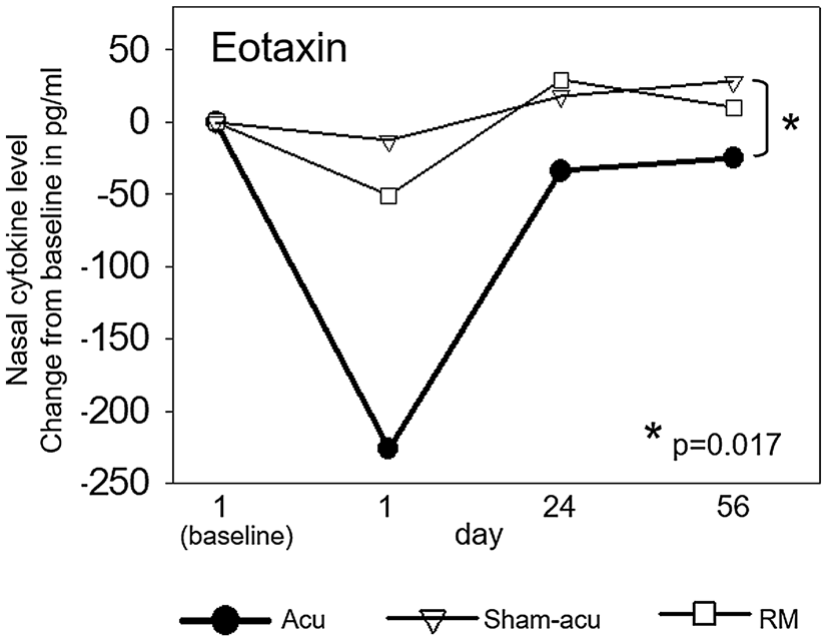
Median change to baseline nasal concentration of eotaxin throughout intervention in acupuncture (bold black line), sham acupuncture (white triangles) and RM (white squares). Directly after the first treatment, all patients had a lower nasal concentration of eotaxin compared to baseline, however particular after real acupuncture. At the subsequent two time-points, the nasal eotaxin level had increased. After 8 weeks of intervention, patients treated by real acupuncture had a significantly lower nasal eotaxin concentration than patients having undergone sham acupuncture

The characteristic trend, which was registered in the nasal concentration of several unspecific pro-inflammatory cytokines mentioned above, could be seen regarding eotaxin, a biomarker of eosinophilia, too (see Table 2). Directly after the first treatment, as displayed in Fig. 3, the nasal concentration of eotaxin was reduced in all treatment arms, however, particularly after real acupuncture. At the subsequent time-points of measurements, the nasal level of eotaxin increased again. After 12 sessions of real acupuncture, the final eotaxin level was still lower than at baseline, whereas both alternative treatments, sham acupuncture or RM alone, led to eotaxin concentrations rather equal to baseline. As seen in IL-1β, the difference in final nasal eotaxin concentration was statistically significant between real acupuncture and sham acupuncture (*p* = 0.017).

**Table 2:** Median changes from baseline nasal concentration of various mediators at day 1, 28 and 56, respectively, and comparison of these median changes between the three treatment arms

Median change from baseline nasal concentration and range							Friedman Analysis		Comparison of median changes from baseline nasal concentration between the groups (Rank Sum Test)					
Treatment	Day 1		Day 28		Day 56		*p*-value	Cytokine	Acu vs. Sham	*p*-value	Acu vs. RM	*p*-value	RM vs. Sham	*p*-value
Acu	− 226	(− 394 to + 63)	− 34	(− 353 to + 54)	− 25	(− 334 to + 119)	*0.122*	**Eotaxin**	day 1	*0.174*	day 1	*0.148*	day 1	*0.435*
Sham-Acu	− 13	(− 61 to 14)	18	(− 44 to 478)	28	(0 to + 177)	*0.367*		day 28	*0.089*	day 28	*0.054*	day 28	*1.0*
RM	− 51	(− 125 to 25)	29	(− 116 to + 142)	10	(− 2 to + 142)	*0.237*		day 56	***0.017***	day 56	*0.079*	day 56	*0.476*
Acu	− 6	(− 50 to + 2)	0	(− 45 to + 54)	0	(− 36 to + 8)	***0.048***	**IL-1β**	day 1	*0.317*	day 1	*0.877*	day 1	*0.833*
Sham-Acu	− 1	(− 6 to 0)	0	(− 6 to + 7)	19	(0 to + 398)	*0.093*		day 28	*0.734*	day 28	*0.571*	day 28	*0.841*
RM	− 4	(− 1123 to + 4)	− 1	(− 1121 to + 83)	− 6	(− 1120 to + 10)	*0.431*		day 56	***0.035***	day 56	*1.0*	day 56	*0.114*
Acu	− 115	(− 505 to + 48)	− 34	(− 498 to + 526)	− 45	(− 345 to + 191)	*0.266*	**MIP-1β**	day 1	*0.429*	day 1	*0.375*	day 1	*0.833*
Sham-Acu	− 9	(− 76 to + 9)	− 19	(− 48 to + 100)	17	(− 6 to + 156)	***0.008***		day 28	*0.562*	day 28	*0.712*	day 28	*1.0*
RM	− 27	(− 2839 to + 97)	− 3	(− 2671 to + 133)	29	(− 2848 to + 132)	*0.431*		day 56	*0.174*	day 56	*0.762*	day 56	*0.914*
Acu	− 45	(− 290 to + 86)	33	(− 533 to + 437)	12	(− 294 to + 109)	*0.301*	**MCP-1**	day 1	*0.650*	day 1	*0.483*	day 1	*0.435*
Sham-Acu	− 38	(− 101 to + 13)	− 2	(− 101 to + 43)	25	(− 63 to + 212)	*0.367*		day 28	*0.428*	day 28	*0.497*	day 28	*0.690*
RM	− 8	(− 841 to + 23)	2	(− 757 to + 82)	18	(− 807 to + 34)	*0.431*		day 56	*0.543*	day 56	*0.856*	day 56	*0.476*
Acu	− 1192	(− 4730 to + 251)	1083	(− 3699 to + 8974)	49	(− 3250 to + 2429)	***0.002***	**IL-8**	day 1	*0.268*	day 1	*0.464*	day 1	*0.724*
Sham-Acu	− 424	(− 1411 to + 49)	17	(− 1272 to + 4542)	642	(− 602 to 15,287)	** < *0.001***		day 28	*0.257*	day 28	*0.119*	day 28	*0.537*
RM	− 285	(− 12,586 to + 434)	− 267	(− 10,535 to + 1173)	632	(− 12,380 to + 1466)	*0.273*		day 56	*0.147*	day 56	*0.856*	day 56	*0.610*
Acu	− 16	(− 343 to + 54)	0	(− 281 to + 114)	9	(− 190 to + 124)	*0.209*	**IL-7**	day 1	*1.00*	day 1	*0.591*	day 1	*0.524*
Sham-Acu	− 23	(− 41 to + 7)	0	(− 25 to + 59)	16	(− 7 to 119)	*0.093*		day 28	*0.777*	day 28	*0.777*	day 28	*0.690*
RM	− 2	(− 202 to + 30)	29	(− 182 to + 40)	12	(− 191 to + 49)	*0.069*		day 56	*0.512*	day 56	*0.903*	day 56	*0.476*
Acu	− 8329	(− 27,749 to + 27,665)	− 1787	(− 30,048 to + 36,633)	− 1463	(− 28,982 to + 41,551)	*0.836*	**IP-10**	day 1	*0.225*	day 1	*0.263*	day 1	*0.724*
Sham-Acu	− 958	(− 5795 to + 551)	280	(− 79 to 27,847)	1481	(− 3470 to + 73,066)	*0.367*		day 28	*0.308*	day 28	*0.650*	day 28	*0.421*
RM	− 511	(− 36,836 to + 8436)	− 1087	(− 41,006 to + 845)	− 3827	(− 34,996 to + 2631)	*0.931*		day 56	*0.174*	day 56	*0.762*	day 56	*0.257*

Statistically significant *p*-values (< 0.05) revealed by intra-group comparison (Friedman Analysis) or inter-group comparison (Rank Sum Test) are bold and underlined

The tendency, that most pro-inflammatory cytokine levels were the lowest after real acupuncture and the highest after sham acupuncture, whereas RM let to less or more lower concentrations than sham acupuncture, was accompanied by a similar trend in the quality of life data, as given in Table 3.

**Table 3:** Median score of nasal symptoms ( taken from the Rhinitis Quality of Life Questionnaire) at baseline and at the end of the study in all 26 patients who had completely answered the questionnaire at the study center in Munich

Treatment	Rhinitis Quality of Life Questionnaire (RQLQ) Nasal symptoms, Score			*p*-value
	Baseline	Day 56	Median change from baseline	
Acupuncture, *n* = 14	3.75 (3.0 to 5.75)	1.62 (0.25 to 4.0)	− 2.0 (− 4.25 to + 1.5)	***0.003***
Sham-acupuncture, *n* = 6	3.13 (1.5 to 4.0)	1.88 (1.0 to 3.5)	− 1.25 (− 2.5 to + 0.75)	*0.156*
Rescue medication, *n* = 6	3.25 (1.0 to 5.25)	2.0 (1.5 to 4.5)	− 1.5 (− 3.25 to + 3.5)	*0.438*

All three treatment arms experienced a reduction in nasal symptoms (acupuncture > RM > sham acupuncture). However, only after real acupuncture, the reduction in nasal symptoms was statistically significant

All values—except for the *p*-values—are median with ranges in parentheses

Bold italic indicates the statistically significant *p*-value of 0.003

## Discussion

In the literature, there is an increasing number of studies revealing effectiveness of acupuncture in treating various diseases. Not least, the ACUSAR trial, which the data of this study originate from, could show a significant improvement in disease-specific quality of life and antihistamine use in patients with seasonal allergic rhinitis after 8 weeks of acupuncture compared with sham acupuncture or rescue medication alone [9]. However, the underlying mechanisms still remains speculative. As allergies are caused by an imbalance between Th1- and Th2-cell response, one possible point of action could be a modulation of the Th1/Th2-balance by acupuncture. However, our analysis did not reveal any acupuncture-related significant change in concentrations of cytokines associated to the Th1- or Th2-pathway, neither locally in nasal fluid nor systemically in plasma. When looking into the literature, a decrease in IL-4, IL-5 and IL-13 after acupuncture could be seen in animal experiments, namely in rats suffering from allergic rhinitis [26]. Two studies on humans with AR, however, revealed constant concentrations of IL-4 in the blood [19, 20]. However, there is, to our knowledge, no published study analysing nasal fluid in this context. Concerning total and specific IgE which are considered as representative markers of allergy, too, controversial results have been found [19, 20, 27]. However, even in a study revealing a statistically significant change in specific IgE against mites after acupuncture, the decrease (from 18 to 16 kU/l) is too low to expect a clinical significance of the finding [20].

Also with regard to the Th1-pathway, controversial results can be found in the literature. Whereas gene expression profiles in patients with AR before and after acupoint herbal plaster have shown an up-regulation of the Interferon (IFN)-signalling [28], the concentration of IFN-γ remained constant throughout acupuncture treatment in humans with AR in other trials [19, 20, 27].

One could go into detail and analyse the potential limitations of the various trials in order to rate the validity of each study and its result. However, it appears meaningful to us that acupuncture does not specifically affect the Th1/Th2-pathway, since acupuncture is known to improve many different disease entities suggesting a more general physiological mechanism of acting.

This suggestion is further underlined by our observation that the plasma and nasal level of IL-10, a marker of the Treg-cluster, remained constant despite acupuncture. IL-10 is able to hinder the histamine release of activated mast cells [29] and can be regarded as a biomarker reflecting the effectiveness of the anti-allergic therapy [30]. Our finding that acupuncture improved the disease-specific quality of life without affecting the IL-10 level, can be regarded as further hint that acupuncture does not act as specific anti-allergic therapy, but throughout a different mechanism.

One possible mode of action could be a reduction in unspecific inflammation. Some biomarkers of unspecific inflammation, such as IL-2 and IL-17, remained below the cut-off value, whereas the concentration of TNF-alpha and IL-6 were above the cut-off, but did not show any relevant change in concentration throughout intervention. With respect to IL-6, our finding is confirmed by another study [31].

Regarding all other assessed cytokine profiles, which are related to unspecific inflammation (IL-1β, IL-7, IL-8, IP-10, MCP-1 and MIP-1β), a general trend could be registered: directly after the first session of treatment, a decrease in cytokine level was found compared to baseline, especially after real acupuncture. One potential explanation could be a dilution effect due to a treatment-associated rhinorrhea. On the other hand, a small decrease was also seen in the two other groups—this result might be due to a local irritation of the nasal mucosa by the sampling of nasal fluid twice within a few hours.

Following this initial decrease, concentrations of most pro-inflammatory cytokines increased again and finally reached levels above the baseline value. This general increase during the intervention period is probably due to the ongoing pollen season enhancing the inflammation over time. Furthermore, as most patients were polysensitized to more allergens than birch and grass, most patients will have been exposed to a higher number of relevant allergen sources at the end of the study than in the beginning.

At the latest time point of measurement, after 8 weeks of intervention, the level of pro-inflammatory cytokines was the highest among the sham-acupunctured patients followed by the RM group. Patients who had been treated by real acupuncture for 8 weeks, had the lowest levels of pro-inflammatory cytokines. This partially statistically significant finding could be seen as confirmation that real acupuncture leads to a reduction of unspecific inflammation. As this treatment effect was only seen after real acupuncture, although patients were blinded to real acupuncture and sham acupuncture, this result cannot be regarded as placebo effect. One might speculate that the anti-inflammatory effect of acupuncture is of clinical relevance, as it was accompanied by a statistically significant improvement in the nasal symptom score of RQLQ, which was only experienced by patients receiving acupuncture.

Still, it remains to discuss why patients, who had received sham acupuncture, had higher levels of pro-inflammatory cytokines than patients treated with RM only. This discrepancy is not statistically significant, though it appears relevant as it goes along with the same trend in the quality of life results: in the present sub-study, patients treated by RM solely had a better nasal and overall quality of life compared to sham-acupunctured patients. At first sight, it appeared likely that this discrepancy in the level of inflammatory cytokines and in the quality of life between sham acupuncture and RM alone is due to a discrepant antihistamine use. Antihistamines are an effective drug for reducing inflammation and cytokine release. That is why histamine receptor antagonists are currently widely discussed in the context of COVID-19 as they might reduce the histamine-mediated pulmonary cytokine storm [32]. In the context of AR, it is proven that histamine upregulates the expression of various chemokines including MCP-1 and eotaxin in the nasal mucosa [33]. This increase can be inhibited by a second-generation antihistamine [33, 34], which also reduces the concentration of IP-10, MIP-1β, IL-8 [35] and IL-1β [36]. Indeed, the ACUSAR study had shown that the percentage of patients using RM and the taken dose was higher among patients receiving sham acupuncture than among patients without any acupuncture [37]. However, when focussing on individual patients in our study center, we could not find a clear correlation between rescue medication score and cytokine level.

With regard to non-allergic diseases, several studies could demonstrate an anti-inflammatory effect by acupuncture in animals, for example in rats suffering from peritonitis [38] or M. Alzheimer [39]. Human trials, which investigate a potential anti-inflammatory effect of manual acupuncture, are rare. Nevertheless, in one recent study, acupuncture has been found to reduce the serum concentration of IL-1β in patients suffering from knee osteoarthritis [40]. Furthermore, in the context of the therapeutic effect of acupuncture on type I hypersensitivity itch or histamine-induced itch, it has been speculated that the influence on itch is probably because of effects on inflammation (probably neurogenic inflammation) and probably not specifically antipruritic [41, 42].

With regard to eotaxin, our literature search revealed only one human study demonstrating a constant plasma level of eotaxin after 12 sessions of acupuncture in persistent AR [20] which is in line with our observation.

However, a direct comparison of our findings in nasal fluid with the literature is not possible, as the present study is to our knowledge the first published analysis of nasal fluid in the context of acupuncture in SAR.

There are several limitations in this study: the most relevant limitation is the small sample size of this sub-study, especially in both control groups due to the allocation ratio of the randomisation. Furthermore, the concentration of mediators in nasal secretion varies a lot among individuals prohibiting a comparison of concrete values. Consequently, the use of median change from baseline was required to allow comparability. In addition, the exposure to allergens was not standardized as it did not follow an experimental design, but was caused by natural airborne pollen of which the concentration and composition may differ locally.

Furthermore, the determination of other mediators, especially the local concentration of Substance P and (specific) IgE, would have been interesting. However, the volume of nasal fluid varies individually by a large amount. In most patients, there was too less material for a wider repertoire of measurements, especially as the determination of IgE by ImmunoCAP needs a rather large sample volume.

Another critical point might be that, according to the principle of segmental innervation by Head [43], the skin is linked to the inner organs. Although sham exposure did not include needling in the head and neck region penetrating sham exposure cannot be regarded as complete placebo treatment as an effect on the immune system is not fully excluded.

Despite several limitations, the present data are based on a study design and methodology of very high quality and represent, to our knowledge, the first analysis of nasal fluid in the context of acupuncture treating allergic rhinitis. Due to these advantages, the data are to be considered as an important step towards a better understanding of the acting mechanism of acupuncture in AR.

In the future, further investigations on the potential mechanism of acupuncture are crucial. A promising target might be mediators of the neuroendocrine pathways, as autonomic dysfunction has been shown to play an important role in the induction and aggravation of AR [44]. Furthermore, another sub-study of the ACUSAR trial has shown that acupuncture partially normalized SAR-related alterations of the autonomic function [45]. When focussing on rather unexplored autonomic neuroendocrine processes, Substance P, a pro-inflammatory neuropeptide, might be an interesting target to analyse as it might trigger the acupuncture-induced signalling [46]. Furthermore, Substance P has been deeply investigated in the context of idiopathic rhinitis in the past [47] and in recent times [48]. However, it might also play an essential role in AR [49].

In sum, our sub-study of the large ACUSAR trial reveals an inhibition of nasal pro-inflammatory cytokines in patients with SAR during real acupuncture. This anti-inflammatory effect by real acupuncture was not achieved by sham acupuncture. An immunological effect on the Th1/Th2 balance, however, could not be seen. Consequently, the effectiveness of acupuncture in the treatment of allergies does not seem to be caused by a specific immunologic anti-allergic mechanism. Instead, an unspecific anti-inflammatory effect appears to be more likely. It appears conceivable, that an anti-inflammatory effect might even explain the continuing treatment effect by acupuncture, as seen in the ACUSAR trial even in the subsequent pollen season although no further treatment had been applied after the end of the preceding pollen season.

The presented sub-study emphasizes the conduction of larger trials on this mechanism of acupuncture, especially as an anti-inflammatory effect might further explain the positive treatment effect of acupuncture on various non-allergic diseases.

## Supplementary Information

Below is the link to the electronic supplementary material.

Supplementary file1 (DOCX 35 kb)
