# Supplementary material for: Acupuncture and its effect on cytokine and chemokine profiles in seasonal allergic rhinitis: a preliminary three-armed, randomized, controlled trial
Source: Eur Arch Otorhinolaryngol. 2022 Mar 17;279(10):4985–95. doi: 10.1007/s00405-022-07335-5 (PMC8929452; doi:10.1007/s00405-022-07335-5)
Supplement: Supplementary file 1 — Supplementary file1 (DOCX 35 kb) [file 405_2022_7335_MOESM1_ESM.docx]

**Supplement Table 1: Plasma concentrations (pg/ml) given in median and range (min to max) for all biomarkers with plasma levels above the cut-off.**

| **Cytokine** | **Treatment** | **Cytokine concentation and range (pg/ml)** | | | **Friedman**  **analysis** |
| --- | --- | --- | --- | --- | --- |
|  |  | **baseline** | **day 28** | **day 56** | ***p-value*** |
|  |  |  |  |  |  |
| **Eotaxin** | **Acupuncture** | 62.4 (29 to 98) | 64.3 (31 to 93) | 59.5 (30 to 82) | *0.264* |
|  | **Sham-Acu** | 49.2 (21 to 114) | 31.3 (27 to 64) | 43.2 (24 to 110) | *0.522* |
|  | **Rescue Med.** | 46.9 (31 to 59) | 33.2 (23 to 44) | 42.4 (24 to 62) | *0.124* |
|  |  |  |  |  |  |
| **IP-10** | **Acupuncture** | 444.9 (295 to 865) | 378.2 (302 to 967) | 447.1 (295 to 947) | *0.144* |
|  | **Sham-Acu** | 334.4 (169 to 510) | 363.8 (273 to 499) | 346.4 (160 to 498) | *0.367* |
|  | **Rescue Med.** | 346.7 (250 to 4130) | 348.9 (288 to 488) | 379.3 (291 to 467) | *0.954* |
|  |  |  |  |  |  |
| **IFNγ** | **Acupuncture** | 2.3 (0 to 17) | 2.3 (0 to 8) | 3.8 (0 to 11) | *0.388* |
|  | **Sham-Acu** | 1.7 (0 to 9) | 1.6 (0 to 6) | 2.3 (1 to 4) | *0.954* |
|  | **Rescue Med.** | 1.3 (0 to 5) | 0.0 (0 to 2) | 0,0 (0 to 1) | *0.691* |
|  |  |  |  |  |  |
| **IL-6** | **Acupuncture** | 1.8 (0 to 4) | 1.5 (0 to 3) | 1.5 (0 to 3) | *0.649* |
|  | **Sham-Acu** | 2.5 (1 to 6) | 2.0 (1 to 3) | 1.5 (0 to 4) | *0.954* |
|  | **Rescue Med.** | 2.1 (0 to 6) | 0.3 (0 to 1) | 0.3 (0 to 2) | *0.691* |
|  |  |  |  |  |  |
| **IL-7** | **Acupuncture** | 4.7 (0 to 28) | 4.7 (0 to 28) | 4.7 (0 to 28) | *0.839* |
|  | **Sham-Acu** | 2.3 (0 to 36) | 4.7 (0 to 23) | 2.3 (0 to 18) | *0.954* |
|  | **Rescue Med.** | 18.2 (0 to 40) | 4.7 (0 to 36) | 18.2 (0 to 18) | *0.431* |
|  |  |  |  |  |  |
| **IL-8** | **Acupuncture** | 4.5 (1 to 17) | 4.5 (0 to 15) | 6.6 (2 to 19) | *0.258* |
|  | **Sham-Acu** | 5.2 (0 to 17) | 4.5 (2 to 7) | 3.8 (0 to 9) | *0.954* |
|  | **Rescue Med.** | 3.4 (0 to 7) | 1.6 (0 to 9) | 3.1 (0 to 6) | *1.0* |
|  |  |  |  |  |  |
| **MCP-1** | **Acupuncture** | 51.8 (26 to 70) | 56.8 (17 to 69) | 48.2 (19 to 85) | *0.587* |
|  | **Sham-Acu** | 52.3 (31 to 68) | 51.8 (35 to 53) | 51.5 (28 to 68) | *0.954* |
|  | **Rescue Med.** | 44.1 (19 to 66) | 40.8 (17 to 47) | 38.9 (23 to 49) | *0.954* |
|  |  |  |  |  |  |
| **MIP-1β** | **Acupuncture** | 17.7 (8 to 46) | 15.1 (5 to27) | 18.4 (8 to 31) | *0.186* |
|  | **Sham-Acu** | 12.4 (0 to 35) | 15.5 (8 to 18) | 10.4 (2 to 19) | *0.431* |
|  | **Rescue Med.** | 17.1 (5 to 42) | 11.2 (6 to17) | 12.4 (6 to 21) | *0.954* |
|  |  |  |  |  |  |
| **TNFα** | **Acupuncture** | 9.7 (4 to 25) | 17.5 (0 to 29) | 17.5 (0 to 34) | *0.717* |
|  | **Sham-Acu** | 9.7 (10 to 52) | 15 (0 to 20) | 9.7 (10 to 15) | *0.691* |
|  | **Rescue Med.** | 10.9 (0 to 42) | 20 (7 to 29) | 6.8 (0 to 25) | *0.182* |
|  |  |  |  |  |  |
| **IL-4, IL-5, IL-13, IL-2, IL-12, IL-10, IL-1β, IL-17: below the cut-off value** | | | | |  |
|  | | | | |  |

**Supplement Table 2: Median changes from baseline plasma concentration of various mediators at day 28 and 56, respectively, and comparison of these median changes between the three treatment arms.**

|  | **Median change from baseline plasma concentration and range (pg/ml)** | |  | **Comparison of median changes from baseline plasma concentrations between the groups (Rank Sum Test)** | | | | | |
| --- | --- | --- | --- | --- | --- | --- | --- | --- | --- |
| **Treatment** | **day 28** | **day 56** | **Cytokine** | **Acu vs. Sham** | *p-value* | **Acu vs. RM** | *p-value* | **RM vs. Sham** | *p-value* |
|  |  |  |  |  |  |  |  |  |  |
| **Acu** | 1 (-25 to +41) | 1 (-13 to +17) | **Eotaxin** | day 28 | *0.379* | day 28 | *0.151* | day 28 | *0.421* |
| **Sham-Acu** | -4 (-23 to +11) | -5 (-11 to +37) |  | day 56 | *0.815* | day 56 | *0.712* | day 56 | *0.931* |
| **RM** | -8 (-18 to +2) | -2 (-8 to +7) |  |  |  |  |  |  |  |
|  |  |  |  |  |  |  |  |  |  |
| **Acu** | -85 (-362 to +547) | -25 (-109 to +153) | **IP-10** | day 28 | *0.071* | day 28 | *0.547* | day 28 | *0.548* |
| **Sham-Acu** | 7 (-16 to +212) | -2 (-42 to +71) |  | day 56 | *0.374* | day 56 | *0.225* | day 56 | *0.329* |
| **RM** | -38 (-3782 to +238) | 41 (-67 to +105) |  |  |  |  |  |  |  |
|  |  |  |  |  |  |  |  |  |  |
| **Acu** | 0 (-8 to +1) | 1 (-5 to +4) | **IFN-γ** | day 28 | *0.853* | day 28 | *0.889* | day 28 | *1.0* |
| **Sham-Acu** | 0 (-9 to 6) | 0 (-7 to +3) |  | day 56 | *0.373* | day 56 | *0.268* | day 56 | *0.931* |
| **RM** | 0 (-2 to +1) | 0 (-2 to +1) |  |  |  |  |  |  |  |
|  |  |  |  |  |  |  |  |  |  |
| **Acu** | 0 (-3 to +2) | 0 (-3 to +1) | **IL-6** | day 28 | *0.677* | day 28 | *0.487* | day 28 | *0.690* |
| **Sham-Acu** | -1 (-5 to +1) | 0 (-6 to +1) |  | day 56 | *1.00* | day 56 | *0.958* | day 56 | *0.931* |
| **RM** | -1 (-5 to +1) | -1 (-2 to +1) |  |  |  |  |  |  |  |
|  |  |  |  |  |  |  |  |  |  |
| **Acu** | 0 (-28 to +28) | 0 (-14 to +28) | **IL-7** | day 28 | *0.308* | day 28 | *0.354* | day 28 | *0.056* |
| **Sham-Acu** | 0 (-31 to 0) | 0 (-36 to +18) |  | day 56 | *0.925* | day 56 | *0.331* | day 56 | *0.171* |
| **RM** | 5 (0 to 18) | 18 (-18 to +18) |  |  |  |  |  |  |  |
|  |  |  |  |  |  |  |  |  |  |
| **Acu** | 0 (-7 to +9) | 2 (-1 to +15) | **IL-8** | day 28 | *0.247* | day 28 | *0.967* | day 28 | *0.329* |
| **Sham-Acu** | -3 (-12 to +3) | -1 (-13 to +2) |  | day 56 | *0.083* | day 56 | *0.482* | day 56 | *0.310* |
| **RM** | 0 (-3 to +6) | 1 (-2 to 5) |  |  |  |  |  |  |  |
|  |  |  |  |  |  |  |  |  |  |
| **Acu** | -2 (-27 to +15) | -6 (-16 to +15) | **MCP-1** | day 28 | *0.817* | day 28 | *0.487* | day 28 | *0.690* |
| **Sham-Acu** | -1 (-16 to +9) | -2 (-10 to +7) |  | day 56 | *0.637* | day 56 | *0.792* | day 56 | *0.931* |
| **RM** | -3 (-25 to +4) | 4 (-16 to +6) |  |  |  |  |  |  |  |
|  |  |  |  |  |  |  |  |  |  |
| **Acu** | -2 (-32 to +13) | 0 (-10 to +4) | **MIP-1β** | day 28 | *0.963* | day 28 | *0.890* | day 28 | *0.841* |
| **Sham-Acu** | -3 (-16 to +4) | -3 (-15 to +2) |  | day 56 | *0.281* | day 56 | *0.635* | day 56 | *0.537* |
| **RM** | 0 (-33 to +8) | -2 (-6 to +4) |  |  |  |  |  |  |  |
|  |  |  |  |  |  |  |  |  |  |
| **Acu** | 2 (-16 to +26) | 9 (-20 to 16) | **TNF-α** | day 28 | *0.331* | day 28 | *0.579* | day 28 | *0.421* |
| **Sham-Acu** | -5 (-32 to +5) | 0 (-37 to +5) |  | day 56 | *0.146* | day 56 | *0.370* | day 56 | *0.792* |
| **RM** | 5 (-13 to +20) | -4 (-7 to +11) |  |  |  |  |  |  |  |
|  |  |  |  |  |  |  |  |  |  |
